# Reconfigurable beam system for non-line-of-sight free-space optical communication

**DOI:** 10.1038/s41377-019-0177-3

**Published:** 2019-07-24

**Authors:** Zizheng Cao, Xuebing Zhang, Gerwin Osnabrugge, Juhao Li, Ivo M. Vellekoop, Antonius M. J. Koonen

**Affiliations:** 1 Institute for Photonic Integration, Eindhoven University of Technology, PO Box 513, 5600 MB Eindhoven, the Netherlands; 2 Faculty of Science and Technology, University of Twente, P.O. Box 217, 7500 AE Enschede, the Netherlands; 3 State Key Laboratory of Advanced Optical Communication Systems and Networks, Peking University, Beijing, 100871 China

**Keywords:** Optics and photonics, Optical techniques

## Abstract

In this paper, we propose a reconfigurable beam-shaping system to permit energy-efficient non-line-of-sight (NLOS) free-space optical communication. Light is steered around obstacles blocking the direct communication pathway and reaches a receiver after reflecting off of a diffuse surface. A coherent array optical transmitter (CAO-Tx) is used to spatially shape the wavefront of the light incident on a diffuse surface. Wavefront shaping is used to enhance the amount of diffusely reflected light reaching the optical receiver. Synthetic NLOS experiments for a signal reflected over an angular range of 20° are presented. A record-breaking 30-Gbit/s orthogonal frequency-division multiplexing signal is transmitted over a diffused optical wireless link with a >17-dB gain.

## Beam shaping: Eliminating blind spots from optical data streams

A new optical wireless communication setup can steer light around obstacles by using liquid crystal-based spatial light modulators to focus on diffuse reflections from ceilings and walls. Light-based systems for sending information are normally limited by direct line-of-sight requirements between transmitter and receiver stations. Zizheng Cao from Eindhoven University of Technology in the Netherlands and colleagues now report a protocol for achieving data rates of 30 gigabyte per second even with blocked sightlines. The team’s approach uses a spatial light modulator to direct the random, speckle-like patterns of scattered light from a wall (emulated by sandblasted aluminum reflector) towards a desired non-line-of-sight user. An algorithm then optimizes phase patterns until the transmitted light intensity is maximized along a free-space pathway around any barriers.

## Introduction

Over the last 10 years, wireless traffic has greatly increased, particularly in indoor scenarios. The number of connected devices is predicted to exceed 50 billion by 2020^1^. To satisfy the growing demand for faster and better wireless communication, two main technologies have been extensively developed: radio wireless communication and optical wireless communication (OWC)^2,3^. Radio wireless communication, especially Wi-Fi, is ubiquitous in both private homes and public spaces. Thus far, the latest 802.11ac IEEE standard allows for a single stream speed up to 866 Mbit/s in the 5-GHz band^4^. Moreover, the latest super Wi-Fi is aimed at providing 7-Gbit/s wireless connection by using the 60-GHz spectrum^5^. Nevertheless, with ever-increasing demand, the limited bandwidth allocated to radio communication will be exhausted very quickly. When too many users are connected to the same access point, Wi-Fi quickly overloads and becomes sluggish. Wireless communication by means of light (a.k.a. OWC)^6–10^ can bring a breakthrough in communication capabilities, both in terms of ultra-high capacity per user and in terms of electromagnetic interference-free communication. OWC has a wealth of additional unlicensed optical spectra and enables the creation of smaller and intense smart communication cells, which can offload heavy data traffic from congested radio wireless networks^2,10^. Currently, there are two main methods for indoor OWC, i.e., low-cost visible light communication^8,11–14^ and broadband beam-steered infrared light communication^7,15–17^, for which capacities >10 Gbit/s^14^ and >400 Gbit/s^7^ have been achieved in the laboratory, respectively.

However, one fundamental challenge for OWC arises when the direct pathway between the transmitter and the receiver is obstructed by an obstacle. In indoor applications, a non-line-of-sight (NLOS) link could potentially be established using light that is diffusely reflected off a scattering material (e.g., the ceiling or a wall). When an optical beam is incident on a rough surface, the light is scattered in a disordered manner, resulting in a near-isotropic, speckled intensity distribution of the diffusely reflected light. Therefore, at the receiver end, the intensity of the diffused light is inherently much lower than that of a collimated incident light beam arriving directly at the receiver. The proposed solutions to this issue usually do not address the diffusion mechanism itself but instead focus on the compensation of diffuse losses by increasing the system power, or they avoid diffuse reflection altogether, i.e., using a near-perfect mirror as a reflector. However, the allowed power is limited by eye-safety regulations, while implementing mirrors is often costly and impractical^9,18^. As a long-standing challenge, such diffuse losses critically hinder the wide application of OWC.

In this paper, we present a novel solution to this challenge. We enhance the intensity of a diffuse NLOS link by means of wavefront shaping^19,20^. Wavefront shaping is a well-known technique in the field of scattering optics with applications in, for instance, deep-tissue microscopy^21,22^, micro-manipulation^23^, and quantum secure authentication^24^. This technique allows light to be focused through and inside opaque materials by controlling the wavefront of the light using a spatial light modulator (SLM), greatly enhancing the light intensity at the desired location (or direction). Here, we use wavefront shaping to establish a diffuse NLOS link by spatially controlling the wavefront of the light incident on a diffuse reflector, maximizing the scattered optical power at an OWC receiver. The diffuse NLOS link is directionally adjustable, which is essential in an indoor beam-steered OWC system. We are the first to introduce wavefront shaping to address NLOS issues in an OWC system to the best of our knowledge. We demonstrate this technique experimentally, which we have dubbed ‘coherent array optical transmitter’ (CAO-Tx). Using the proposed CAO-Tx, a record data rate of 30 Gbit/s is transmitted over a diffuse link with an angular steering range of 20°.

We first explain the operation principle of the CAO-Tx, and then the experimental setup is detailed. The experimental results are shown and analyzed in the next section. Finally, we discuss the practical challenges for future applications. For the readers’ convenience, the acronym list is shown in Table 1.

**Table 1:** Acronym list

OWC	Optical wireless communication
NLOS	Non-line-of-sight
LOS	Line-of-sight
SLM	Spatial light modulator
CAO-Tx	Coherent array optical transmitter
AWG	Arbitrary waveform generator
ECL	External cavity laser
EDFA	Erbium-doped fiber amplifier
PC	Polarization controller
SLM	Spatial light modulator
OLO	Optical local oscillator
BPD	Balanced Photodiodes
VOA	Variable optical attenuator
ADC	Analog-to-digital converter
DAC	Digital-to-analog converter
DPO	Digital phosphor oscilloscope
OFDM	Orthogonal frequency-division multiplexing
16QAM	16-ary quadrature amplitude modulation
FEC	Forward error correction

## Results

### Wavefront shaping to focus a scattered beam

**Fig. 1:**
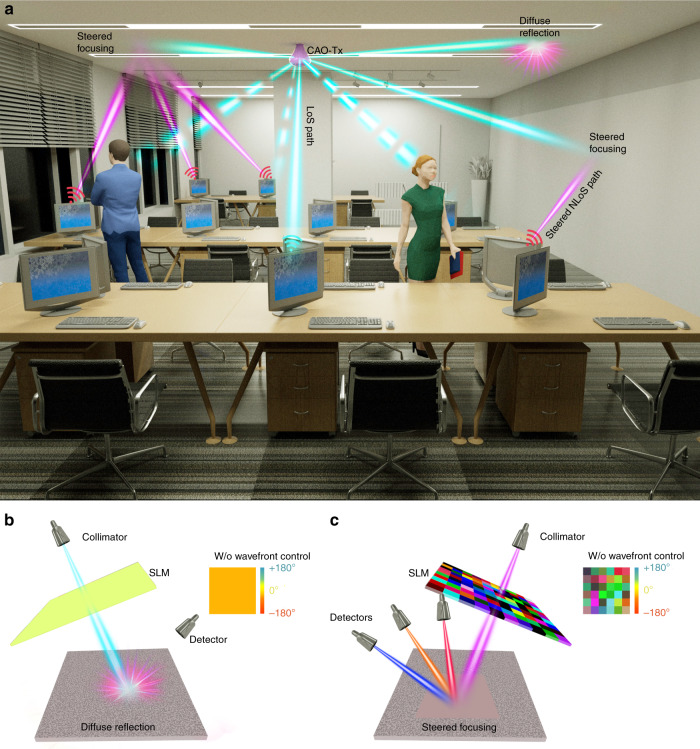
**a** Indoor use case of the coherent array optical transmitter. In the absence of a direct LoS path, diffusely reflected light can be focused to the OWC detector of the wireless device. **b** Basic principles of wavefront shaping: a diffuse reflecting surface is illuminated with a flat wavefront, and the light is randomly scattered in all directions. Only a small fraction of the light reaches the detector. **c** By means of an SLM, the phases of different segments of the incident light are modulated to maximize the intensity at the detector

In Fig. 1a, an indoor use case for the CAO-Tx is depicted. From the access point (the CAO-Tx depicted in Fig. 1a), the narrow light beams with user data are sent to the wireless terminals. Usually, user data are transmitted from an information server to an access point via an indoor fiber network. In some cases, a direct high-speed connection between the CAO-Tx and the terminal can be established via a line-of-sight (LOS) path. However, when the direct pathway is obstructed, the light can instead be directed to a diffuse reflection ceiling or wall to establish an indirect NLOS pathway to the device (right example: diffuse reflection in Fig. 1). However, the light incident on the diffuse reflecting object will be scattered in many different directions, as shown in Fig. 1b. Therefore, the OWC detector at a large distance from the diffuse reflector will only collect a small amount of the diffused light.

To overcome this problem, the CAO-Tx includes an SLM, allowing control over the phase of field *E*_*a*_, which is incident on the diffuse reflecting surface. We subdivide the SLM into *N* different segments, which are all separately controlled. Now, the scattered field reaching the OWC detector of the device can be described by:

1 $$E_b = \mathop {\sum }\limits_a^N t_{ba}E_a$$

where *t*_*ba*_ is an element of the scattering matrix *T*, connecting the *N* number of incident field segments *E*_*a*_ to the detected field *E*_*b*_. Here, all elements of the matrix *T* are assumed to be random complex variables, and as a result, all scattered waves *t*_*ba*_*E*_*a*_ will have a random phase^20,25^. All of these randomly scattered waves interfere, forming a complex intensity pattern known as a speckle pattern.

Assuming that the scattering material does not change during the optimization, we can modulate the phase of *E*_*a*_ to optimize the intensity at the detector. To maximize the intensity $|E_b|^2$, we use the stepwise sequential wavefront-shaping algorithm^20,25^, where the phases of all of the incident field segments are modulated between 0 and 2*π* in a stepwise fashion. As a result, the intensity measured at the detector will vary as a function of *θ*_*a*_, the phase of a single input segment *a*:

2 $$I_b\left( {\theta _a} \right) \equiv \left| {E_b} \right|^2 = \left| {E_{ref} + t_{ba}E_ae^{i\theta _a}} \right|^2$$

with reference field $E_{ref} \equiv \mathop {\sum }\nolimits_{a{\prime} \ne a}^N t_{ba{\prime}}E_{a{\prime}}$. The intensity at the detector is maximized when *E*_*ref*_ and $t_{ba}E_ae^{i\theta _a}$ are in phase, i.e., $\theta _a = arg(E_{ref}) - arg(t_{ba}E_a).$ This procedure is repeated for all input field segments, and finally the optimized phase of all incident field segments is applied to the SLM, resulting in an enhancement of the light intensity at the position of the detector (see Fig. 1c). To enhance the light intensity at a different detector position, the algorithm is performed again to find the new ideal phase pattern.

The diffusely reflected light can be focused to any location in the room as long as the OWC detector is capable of measuring the intensity of the SLM-modulated light during the optimization process. The scattered light can be focused on multiple detectors simultaneously by superimposing multiple ideal phase patterns on the SLM^20,25^. The theoretical intensity enhancement at the detector is independent of the properties of the scattering material. The only limiting factor is the signal-to-noise ratio (SNR) at the receiver^26^. The losses of the diffuse link over large distances can be compensated by optimizing for a larger number of SLM segments since the intensity enhancement increases linearly with *N*^20^. Once the diffuse link with required optical power is obtained using the proposed CAO-Tx, high-speed OWC signals can be transmitted to the receiver.

### The non-line-of-sight optical wireless link

**Fig. 2:**
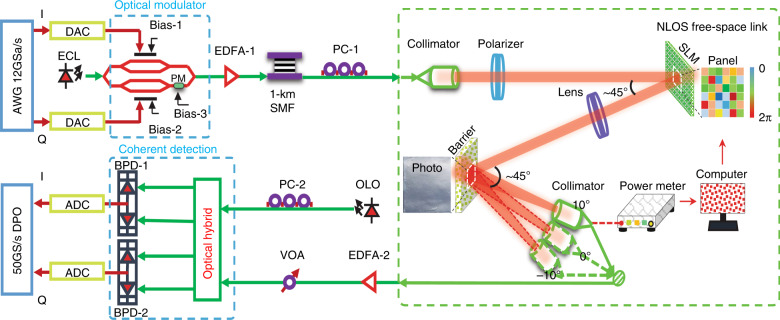
Schematic drawing of the experimental setup. AWG: arbitrary waveform generator; EDFA: Erbium-doped Fiber Amplifier; PC: polarization controller; SLM: spatial light modulator; OLO: optical local oscillator BPD: Balanced Photodiodes; VOA: variable optical attenuator; ADC/DAC: analog-to-digital converter/digital-to-analog converter; DPO: digital phosphor oscilloscope

We proceed to describe the 30-Gbit/s indoor non-line-of-sight beam reconfigurable optical wireless communication system enabled by the CAO-Tx method. Figure 2 depicts the experimental setup and detailed parameters. To match the trend of well-established wireless standards such as IEEE 802.11ac (Wi-Fi) and ITU IMT-Advanced LTE (4G), the widely used orthogonal frequency-division multiplexing (OFDM) signal is adopted. In our experiment, an electrical 30-Gbit/s OFDM signal is generated by an arbitrary waveform generator (AWG). The digital signal processing flow and parameters can be found in S1 in the Supplementary Information. This OFDM signal is then modulated onto an optical carrier via an optical transmitter including an extra cavity laser and an optical IQ modulator. The signal details are presented in the section “Materials and methods”. The optical signal is amplified via an Erbium-doped Fiber Amplifier (EDFA-1). A 1-km bend-insensitive single-mode fiber is used to emulate indoor applications. Afterward, a polarization controller (PC-1) is used to align the polarization of the optical signal to the polarization axis of the SLM before a collimator. The collimated Gaussian beam is then incident on the SLM (HOLOEYE, PLUTO Phase Only SLM; see S4 in the Supplementary Information for more details). The angle between the incident beam and the reflected beam is 45°. To match the size of the Gaussian beam, 1024 × 1024 pixels are activated, which are further grouped into segments of 128 × 128 pixels, yielding a total of 8 × 8 segments. All pixels in a segment are simultaneously modulated from 0 to 2π in increments of π/4. After the phase modulation of these segments, a lens (f = 200 mm) focuses the modulated beam onto a diffuse reflection barrier, which emulates the rough surface (ceiling or wall) in an indoor scenario. Here, two types of scattering samples are tested: a) a Thorlabs polystyrene screen (EDU-VS1/M); b) a sandblasted aluminum film. The angle between the SLM-modulated beam and the normal of the barrier is −22.5°, and we define the principal reflection angle as +22.5° (i.e., the angle of reflection equals the angle of incidence).

To collect the diffusely reflected optical signal, the light is coupled into a fiber using a collimator. The receiving fiber is mounted on a movable stage, allowing the receiving angle and distance to be varied. An optical power meter is used to provide feedback for the wavefront-shaping algorithm to enhance the light intensity at the receiving fiber. The received optical signal is pre-amplified via a second EDFA (EDFA-2) before it is detected by an optical coherent receiver. Finally, the detected signal is sampled by a real-time oscilloscope operating at a 50-GSa/s sampling rate. The sampled signal is then processed through an offline digital signal-processing algorithm, and the binary signal is ultimately recovered.

### Enhancement of received power

**Fig. 3:**
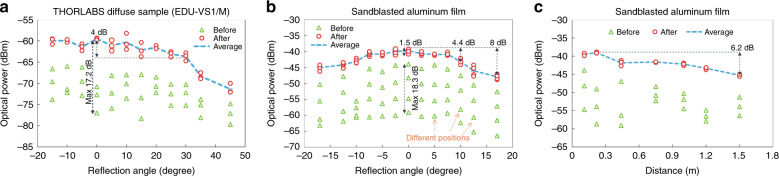
**a**, **b** The measured power versus various reflection angles before and after wavefront shaping by using the THORLABS standard diffuse sample and the sandblasted aluminum, respectively; **c** the measured power versus various distances before and after wavefront shaping

To evaluate the effectiveness of CAO-Tx, we compare cases with and without wavefront shaping. We use the optical power arriving at the receiver (see Fig. 3) as a figure of merit. The enhancement of the received power induced by wavefront shaping is shown in Fig. 3. First, the optimization experiment is performed on the polystyrene screen. To explore the performance of the focusing and the large-scale beam steering (direction tuning), the optical power is measured at a 43-cm distance for an angle ranging from −15° to 45° (offset to the principal reflection angle, similarly hereinafter). Each measurement is performed at 3 different spots on the diffuse reflector, and the results are shown in Fig. 3a. It can be seen that the reflected power slightly decreases with the angle, with a 4-dB half-angular range of ~30°. Through this range, the intensity enhancement remains approximately constant, as expected theoretically, and an average gain of 14 dB is achieved. It can be seen that the initial power fluctuates strongly as the spot on the diffuse reflector is changed. This effect is due to the random speckle distribution of the scattered light. The optimized power does not suffer from this effect, and indeed, the optimized power is largely independent of the position of the spot on the diffuse reflector.

Similar measurements are also performed on the sandblasted aluminum film. The distance between the diffuse reflector and the optical receiver is fixed at 0.11 m. The optical power is measured at an angular offset ranging from −17° to 17°. Each measurement is performed at four different spots on the diffuse reflector. Before wavefront shaping, the received power at the 0° offset intensely fluctuates from −59.1 dBm to −43.9 dBm. In contrast, the received power can always be enhanced to a relatively stable level (1.5-dB fluctuation). An 11.7-dB average enhancement is observed (from −51.5 dBm to −39.8 dBm). When the receiver is located at a dark point (−59.1 dBm), a maximum gain of 18.3 dB could be obtained. This result proves that, even when the receiver is located at a dark spot, the wavefront-shaping algorithm can effectively focus the diffusely reflected light to the receiving collimator. A similar focus enhancement is obtained for other angles, in which the power after wavefront shaping is optimized to ~−42.8 ± 0.8 dBm for a −10° offset and ~−43.8 ± 1.2 dBm for a 10° offset.

Additionally, the distance between the reflector and the receiver is varied from 0.11 m to 1.5 m while keeping the receiver angle fixed at 0°. The measured power-to-distance curves are depicted in Fig. 3c. Again, we notice that, although the signal power decreases with distance, the signal enhancement obtained by wavefront shaping remains approximately constant.

The experimental results of these two diffuse materials prove the effectiveness of power enhancement in a diffuse link achieved by wavefront shaping. Because ceilings and walls are generally diffuse reflectors^15^, this method is expected to be effective as well. Compared with the isotropically scattering polystyrene screen (enhanced power: ~−60 dBm@0.43 m in Fig. 3a), the received power of the sandblasted aluminum has a ~18-dB improvement (~−42.2 dBm@0.44 m in Fig. 3c). Although the angular coverage becomes narrower, the received power is much higher. Therefore, we use the sandblasted aluminum reflector in our data transmission experiment. The detailed scattering response of the two diffuse reflection materials can be found in S2 in the Supplementary Information.

### Record data rate over a diffused link

**Fig. 4:**
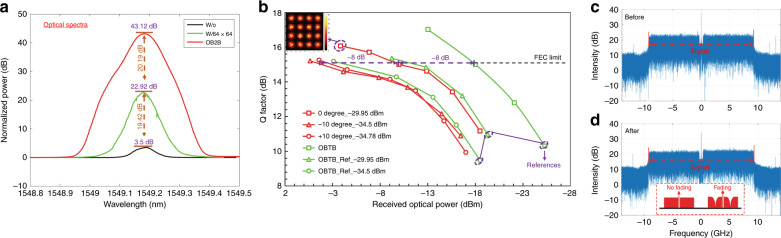
**a** The measured optical spectra; **b** the measured Q factor as a function of the received power; **c**, **d** the RF spectra before (OBTB) and after wavefront shaping

In this experiment, we demonstrate a diffuse NLOS link enabled by CAO-Tx. A record 30-Gbit/s data rate is achieved in a diffused optical wireless communication system. Figure 4a presents the normalized optical spectra (normalized to the same noise level) before and after wavefront shaping to show the optical SNR improvement. The spectrum of the optical back-to-back (OBTB) case without the free-space link serves as a reference with 43.12-dB normalized peak power. The peak values of the spectra before and after wavefront shaping are 3.50 dB and 22.92 dB with a 19.42-dB improvement.

To evaluate the transmission performance of the CAO-Tx link, we measure the Q factor as a function of the received power by adjusting the VOA as shown in Fig. 4b. Here, the Q factor is defined as the electrical SNR^27^. The 8 × 8 segments are further divided into 16 × 16 segments to achieve a higher power gain for the transmission. Compared to the incident power to the reflector (~10 dBm), the diffuse reflection sample in our experiment introduces a >60-dB loss (−50.9 dBm at the received collimator) at 0° offset, which greatly reduces power efficiency. After the wavefront shaping, the power can be enhanced to −29.9 dBm with a 20.9-dB gain. When the angle is shifted to ± 10°, the power is enhanced from −54.40 dBm and −52.10 dBm to −34.50 dBm and −34.78 dBm, respectively. Except for the curve of the default OBTB case (13.7 dBm after fiber), two more reference curves are measured for the OBTB cases, but with the power (after fiber) attenuated to −29.9 dBm (0°) and −34.5 dBm (±10°). We assess power penalties at the forward error correction (FEC) threshold of 3.8 × 10^−3^ (Q = 15.17 dB). The power penalties between the focusing cases and their references (Reference 0° and Reference ±10°) are less than 1.5 dB. This suggests that the diffuse NLOS link does not introduce notable impairment. The power penalty between the case of 0° and the cases of ±10° is 8 dB, which is the same as the penalty between the OBTB case and the cases of Reference ±10°. The 4.6-dB power difference results in an 8-dB power penalty when the received power is low (~−30 dBm). This suggests that the improvement of received power by CAO-Tx is critically important for diffuse NLOS links. Moreover, the received RF spectra generated from the digital Fourier transform of the sampled OFDM signal for the OBTB case (without the free-space link) and the diffuse focused case are shown in Fig. 4c, d, respectively. For wireless communication, a significant limit in the NLOS scenario is frequency fading, as depicted in the inset in Fig. 4d. Typical frequency spectra with (w/) and without (w/o) fading are located on the left side and the right side, respectively. In a fading spectrum, the faded parts usually cause serious inter-symbol interference in the time domain. Such interference plays a major role for system performance degradation, rather than the low received power^28–30^. Compared with the OBTB case (shown in Fig. 4c), no frequency fading is found for the focusing case (shown in Fig. 4d). This absence of frequency fading can be attributed to the limited illuminated area on the diffuse reflector, causing negligible multiple path delays. The shaped wavefront is projected to a spot with a diameter of ~1.5 mm. Consequently, the time delay between the shortest and longest path from the transmitter cannot exceed 10 ps. For the OFDM signal used in our experiment, its cyclic prefix length is 1.333 μs, which is much larger than the maximum time delay (10 ps). Therefore, inter-symbol interference is not a limiting factor in the proposed system. Detailed analysis of the link performance is presented in S3 in the Supplementary Information.

## Discussion

### The angular range of beam focusing/steering

In our data transmission experiment, we demonstrated the focusing of diffusely reflected light over an angular range of 20° using a sandblasted aluminum reflector. To achieve a larger angular range, we may either select a proper incident angle on the diffuse reflector through a mechanical scheme or use a more isotropically scattering sample. Using a more isotropically scattering sample, such as the polystyrene screen (Thorlabs EDU-VS1/M) or a white painted wall, will greatly extend the angular range of our method. However, a larger angular coverage will also result in a higher loss of optical power. The detailed coverage measurement is analyzed in S2 in the Supplementary Information.

### Focus enhancement

The fundamental limit here is the decrease of the light intensity as the detector is moved further away from the diffuse reflecting surface. The decrease in light intensity can be compensated by optimizing for more SLM segments because the focus enhancement is proportional to *N* ^25^ Recently, intensity enhancements as high as a factor of 100,000 have been reported^31^. Furthermore, the distance from the surface to the receiver can be shortened by projecting the SLM pattern on a reflecting surface close to the terminal devices. It is worth noting that the focus enhancement enabled by wavefront shaping will be roughly equal for both types of diffuse reflection materials, as is experimentally demonstrated in Fig. 3a, b.

### Adaption to environment variation

If the surface structure of the scattering ceiling or wall were to change, or any other change in the free-space path, such as air flow or dust particles, were to occur, a new SLM phase pattern can be updated using the wavefront shaping algorithm to re-establish a new NLOS link.

### The speed of beam focusing/steering

Separately controlling more SLM segments will increase the optimization time. In our experiment, the wavefront optimization of 8 × 8 segments, to enhance the optical power at the optical receiver, required ~400 s to complete. This optimization time is too long for most practical applications; however, by efficiently synchronizing the spatial light modulator and the detector, this process can become significantly faster. Additionally, here we used a liquid-crystal SLM with a maximum updating rate of 60 Hz, whereas alternatively, a digital mirror device could be used to modulate the wavefront of the light, which can be up to 3 orders of magnitude faster. Blochet et al. were able to optimize wavefronts at a rate of 4.1 kHz (0.244 ms per SLM segment)^32^. The potential of realizing the higher gain and faster optimization could meet the requirements of indoor applications. In indoor applications, diffuse reflectors (the ceiling and walls) are usually stationary. Therefore, pre-scanning can help accelerate the beam focusing/steering process.

### Optical spectral region for the proposed scheme

Currently, the spectral region of our method is limited by the optical components, such as the SLM, the collimators and so on. In our experiment, the working spectral regions of these components are limited to a wavelength range of 1520–1620 nm. However, wavefront shaping has been widely investigated for visible and near-infrared light applications^25^. Our experimental setup can therefore easily be adjusted to work for a broader range of laser wavelengths.

### Scaling up the data rate

The net data rate of 30 Gbit/s can be further improved by using greater bandwidth or/and more advanced modulation formats. In our experiment, the modulated laser light had a bandwidth of ~9.23 GHz. This is far below the upper limit on the bandwidth, which depends on the amount of time a light pulse spends in a scattering medium^19^. For strongly scattering samples, this time is on the order of picoseconds^33^, which means that a modulated beam with a bandwidth of 100 GHz can still be efficiently focused without a large reduction in enhancement.

### Implementation of an optical receiver

As for the transceiver, the combination of IQ modulation and coherent detection is introduced to double the spectrum efficiency and raise detecting sensitivity. In practical applications, any modulating/detecting type is allowed as long as it can meet the requirements, such as cost, power responsivity, and available spectrum resource. The cost of coherent detection can be dramatically reduced by photonic integrated circuit technology for future applications^34^.

In summary, we have proposed a novel method for optical wireless communication for non-line-of-sight data transmission. By spatially modulating the light incident on a rough ceiling/wall, the CAO-Tx is used to focus the diffusely reflected light to the OWC receiver. The focusing capability of a diffusely reflected beam at distances of 0.11 m to 1.5 m is experimentally measured. A record-breaking 30-Gbit/s OFDM signal is transmitted over an indoor diffuse non-line-of-sight link with a >17-dB gain, in an angular range of 20°, and over a distance of 110 mm from the diffuse reflector. The practical issues, such as the operation spectrum, coverage, environment adaption, focusing speed, higher data rate and implementation of receivers, are discussed. We believe that this method, which breaks the non-line-of-sight limitation of optical wireless communication, will provide a new direction for this field.

## Materials and methods

### The transmitter of optical coherent OFDM

An electrical OFDM signal is generated by an arbitrary waveform generator (AWG) with a 12-GSa/s sampling rate. The bandwidth is ~9.2 GHz with 16-ary quadrature amplitude modulation (16QAM), and the net bit rate is 30 Gbit/s. The detailed calculation of the net bit rate is presented in S1 in the Supplementary Information. Through an optical IQ modulator, the OFDM signal is modulated onto an optical carrier provided by an external cavity laser (ECL). Here, the optical spectrum-efficient quadrature amplitude modulation is employed. The in-phase (I) and quadrature (Q) components are separately modulated onto the optical carrier enabled by 3 bias voltages, in which Bias-1 and Bias-2 are used for carrier-suppression modulation^35^ and Bias-3 is adjusted to obtain a 90° phase shift to generate the quadrature carrier. The central wavelength of the optical carrier could be flexibly set.

### The receiver of optical coherent OFDM

The coherent detection is employed to obtain higher responsivity and spectrum efficiency^36^. A variable optical attenuator (VOA) is placed between the EDFA-2 and the receiver to adjust the received power. An ECL with 14-dBm power is used as an optical local oscillator (OLO). A polarization controller (PC-2) is utilized to align the signal polarization and the OLO polarization. Through the optical hybrid component, the four outputs are detected by two balanced photodiodes. The phase differences between the signal and OLO of the four outputs (top-to-bottom) are 0°, 180°, 90°, and 270°, respectively^36^.

## Supplementary information

Supplemental Material

Authoriaztion from the copyright holder

## References

[1] NCAT. Growth In The Internet of Things. at www.ncta.com/chart/growth-in-the-internet-of-things#.WsSRvubTQ00.link.

[2] Koonen T (2018). Indoor optical wireless systems: technology, trends, and applications. J. Light. Technol..

[3] Wang CX (2014). Cellular architecture and key technologies for 5G wireless communication networks. IEEE Commun. Mag..

[4] CISCO. 802.11ac: The Fifth Generation of Wi-Fi. (CISCO, 2018).

[5] IEEE. Draft Standard for Information technology—Telecommunications and information exchange between systems—Local and metropolitan area networks–Specific requirements Part 11: Wireless LAN Medium Access Control (MAC) and Physical Layer (PHY) Specifications. (IEEE, 2016).

[6] Chan VWS (2006). Free-space optical communications. J. Light. Technol..

[7] Gomez A (2016). Design and demonstration of a 400 Gb/s indoor optical wireless communications link. J. Light. Technol..

[8] Haas HL (2016). What is LiFi?. J. Light. Technol..

[9] Schulz D (2016). Robust optical wireless link for the backhaul and fronthaul of small radio cells. J. Light. Technol..

[10] O’Brien D, Parry G, Stavrinou P (2007). Optical hotspots speed up wireless communication. Nat. Photonics.

[11] Chi YC (2015). Phosphorous diffuser diverged blue laser diode for indoor lighting and communication. Sci. Rep..

[12] Chi YC (2015). 450-nm GaN laser diode enables high-speed visible light communication with 9-Gbps QAM-OFDM. Opt. Express.

[13] Wu TC (2017). Tricolor R/G/B laser diode based eye-safe white lighting communication beyond 8 Gbit/s. Sci. Rep..

[14] Chun H (2016). LED based wavelength division multiplexed 10 Gb/s visible light communications. J. Light. Technol..

[15] Kahn JM, Barry JR (1997). Wireless infrared communications. Proc. IEEE.

[16] Koonen T (2018). High-capacity optical wireless communication using two-dimensional IR beam steering. J. Light. Technol..

[17] Wang K (2012). Experimental demonstration of a full-duplex indoor optical wireless communication system. IEEE Photonics Technol. Lett..

[18] Elgala H, Mesleh R, Haas H (2011). Indoor optical wireless communication: potential and state-of-the-art. IEEE Commun. Mag..

[19] Mosk AP (2012). Controlling waves in space and time for imaging and focusing in complex media. Nat. Photonics.

[20] Vellekoop IM, Mosk AP (2007). Focusing coherent light through opaque strongly scattering media. Opt. Lett..

[21] Horstmeyer R, Ruan HW, Yang CH (2015). Guidestar-assisted wavefront-shaping methods for focusing light into biological tissue. Nat. Photonics.

[22] Tang JY, Germain RN, Cui M (2012). Superpenetration optical microscopy by iterative multiphoton adaptive compensation technique. Proc. Natl Acad. Sci. USA.

[23] Dholakia K, Čižmár T (2011). Shaping the future of manipulation. Nat. Photonics.

[24] Goorden SA (2014). Quantum-secure authentication of a physical unclonable key. Optica.

[25] Vellekoop IM (2015). Feedback-based wavefront shaping. Opt. Express.

[26] Yılmaz H, Vos WL, Mosk AP (2013). Optimal control of light propagation through multiple-scattering media in the presence of noise. Biomed. Opt. Express.

[27] Shieh W, Djordjevic I (2009). OFDM for Optical Communications..

[28] Kalita S (2016). Performance enhancement of a multichannel uncoordinated code hopping DSSS signaling scheme using multipath fading compensator. J. Circuits, Syst. Comput..

[29] Biglieri E, Proakis J, Shamai S (1998). Fading channels: information-theoretic and communications aspects. IEEE Trans. Inf. Theory.

[30] Sahu PP, Singh M (2008). Multi channel frequency hopping spread spectrum signaling using code M-ary frequency shift keying. Comput. Electr. Eng..

[31] Yu HS, Lee K, Park Y (2017). Ultrahigh enhancement of light focusing through disordered media controlled by mega-pixel modes. Opt. Express.

[32] Blochet B, Bourdieu L, Gigan S (2017). Focusing light through dynamical samples using fast continuous wavefront optimization. Opt. Lett..

[33] Johnson PM (2003). Time-resolved pulse propagation in a strongly scattering material. Phys. Rev. E.

[34] Smit M, van der Tol J, Hill M (2012). Moore’s law in photonics. Laser Photonics Rev..

[35] Shieh W, Bao H, Tang Y (2008). Coherent optical OFDM: theory and design. Opt. Express.

[36] Ip E (2008). Coherent detection in optical fiber systems. Opt. Express.

