# Author Correction: Reconfigurable beam system for non-line-of-sight free-space optical communication

**DOI:** 10.1038/s41377-021-00573-y

**Published:** 2021-07-01

**Authors:** Zizheng Cao, Xuebing Zhang, Gerwin Osnabrugge, Juhao Li, Ivo M. Vellekoop, Antonius M. J. Koonen

**Affiliations:** 1 Institute for Photonic Integration, Eindhoven University of Technology, PO Box 513, 5600 MB Eindhoven, The Netherlands; 2 Faculty of Science and Technology, University of Twente, P.O. Box 217, 7500 AE Enschede, The Netherlands; 3 State Key Laboratory of Advanced Optical Communication Systems and Networks, Peking University, 100871 Beijing, China

**Keywords:** Fibre optics and optical communications, Optoelectronic devices and components

Addendum to: *Light: Science & Applications*

10.1038/s41377-019-0177-3

Addendum:

We were recently made aware of the fact that Professor Nabeel A. Riza’s laboratory theoretically proposed earlier a Non-Line of Sight (NLOS) indoor optical wireless communications arrangement with three-dimensional optical beamforming via a spatial light modulator using diffuse reflections from diffuse optical elements^1^. This is exactly the type of arrangement that would benefit greatly from our technique, since the coherent arrayed optical transmitter (CAO-Tx) we proposed makes the reflection focus onto the receiver, rather than diffusely scatter it in all directions. We apologize for the omission of this prior-art.
