# Supplementary material for: Reconfigurable beam system for non-line-of-sight free-space optical communication
Source: Light Sci Appl. 2019 Jul 24;8:69. doi: 10.1038/s41377-019-0177-3 (PMC6804797; doi:10.1038/s41377-019-0177-3)
Supplement: Supplementary file 1 — Supplemental Material [file 41377_2019_177_MOESM1_ESM.pdf]

Reconfigurable beam system for non-line-of-sight free-space optical communication

Authors
Zizheng Cao[1,+,*], Xuebing Zhang[1,+], Gerwin Osnabrugge[2,+], Juhao Li[3], Ivo M. Vellekoop[2,*] and Antonius M. J. Koonen[1,*]

Affiliations
[1] Institute for Photonic Integration, Eindhoven University of Technology, PO Box 513, 5600 MB Eindhoven, the Netherlands
[2] Faculty of Science and Technology, University of Twente, P.O. Box 217, 7500 AE Enschede, the Netherlands
[3] State Key Laboratory of Advanced Optical Communication Systems and Networks, Peking University, Beijing 100871, China
*Author e-mail address: z.cao@tue.nl, i.m.vellekoop@utwente.nl, A.M.J.Koonen@tue.nl.
+These authors contributed equally to this paper.

Supplementary Information

S1 Digital signal processing flow and data rate calculation of the OFDM signal

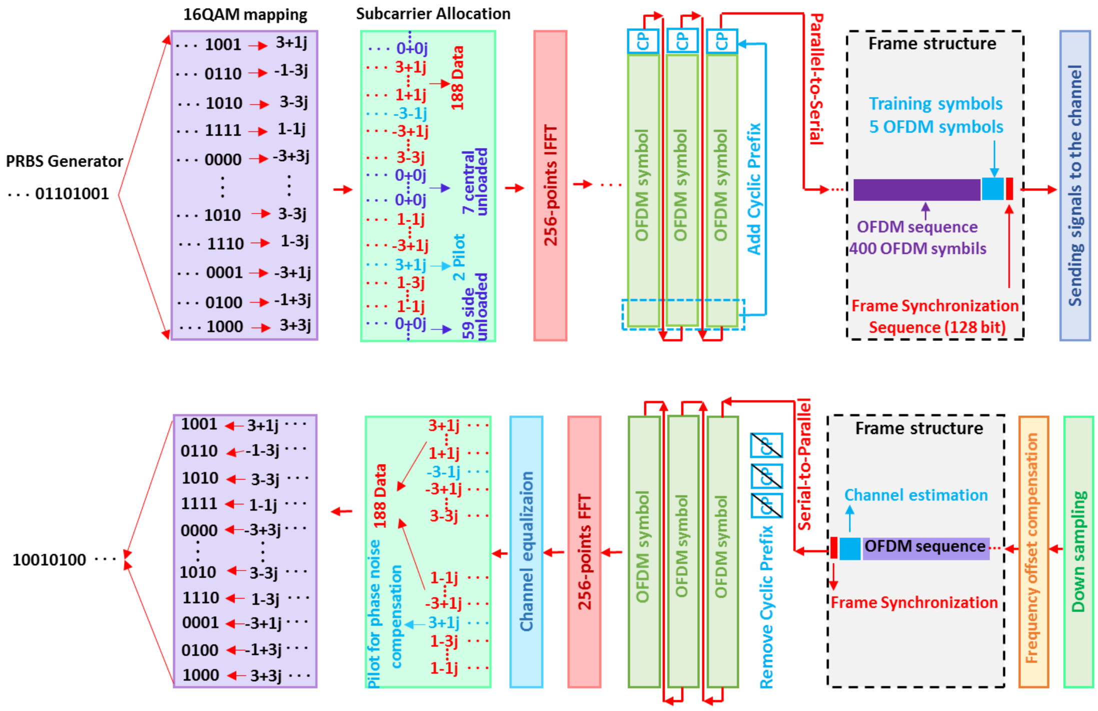

Fig. 1 Offline digital signal processing

A pseudo-random binary sequence (PRBS) is used as data source. The data is mapped to the complex number symbols for the data-loaded subcarriers. Here, 16-ary quadrature amplitude modulation (16-QAM) mapping method is employed due to the capacity and quality of the channel. Then the signal is converted from frequency domain to time domain employing 256-points inverse fast Fourier transform (IFFT), in which 188 subcarriers are loaded with data and 7 subcarriers are unloaded to avoid the DC and low frequency interference. 2 subcarriers are selected as pilots for the phase noise estimation and the other 59 subcarriers on the edge are also data-unloaded. Thus the bandwidth of OFDM-16QAM signal is [1]:

$$12GSa/s \times \ ((188+7+2))/256(\text{data loaded subcarriers}) \approx 9.23\ \text{GHz}\ \ (\text{Double sideband modulation}) \qquad (1)$$

Cyclic prefix (CP) is inserted between two OFDM symbols and the length is 1/16 IFFT size. Parallel-to-Serial conversion is to convert the signal into data sequence. 5 OFDM training symbols are periodically inserted in front of each frame, which is followed by 400 payload symbols. Finally, a 128-bit sequence for time synchronization is added. The net bit rate is:

$$12\ GSa/s \times 186/256\ (\text{data loaded subcarriers}) \times 0.93\ (\text{FEC redundancy}) \times 15/16\ (\text{CP redundancy}) \times 400/405\ (\text{training symbol redundance}) \times 4\text{bit/symbol}\ \approx 30.35\text{Gbit/}s \qquad (2)$$

To demodulate the signal, the same procedure and distortion compensation algorithms are performed in reverse. The received signal is firstly down sampled, then frequency offset needs to be compensated. The frame synchronization is used to extract a frame of OFDM signal for offline processing. The training symbols are used for channel estimation. Afterwards, Serial-to-Parallel conversion, CP removal, FFT are employed to convert the signal into frequency domain and channel equalization is utilized to compensate the channel impairment. The pilot subcarriers are aimed at compensating phase noise. Aided by these procedures, the 16-QAM signal is recovered and then is de-mapped to binary data.

S2 Scattering response of the diffuse reflecting materials

In our diffuse NLOS communication system, the angular coverage mainly depends on the scattering angle range of the diffuse reflector. In the experiments, we tested our method on two different materials: I) a polystyrene screen (Thorlabs EDU-VS1/M) and II) a sandblasted aluminum film. The photos of the scattering samples are shown in Fig. 2 (a). To characterize the scattering response of these materials, we measured the bi-directional reflectance distribution function (BRDF) for an incident angle of -22.5°. The BDRF describes how much optical power is backscattered into a given reflection angle. We measure the BDRFs of the two samples by using an optical power meter with a 3-mm dimeter at a distance of 70 mm from the sample. The laser power incident on the sample was set to 9 dBm at a wavelength of 1550 nm. The measured BRDFs for the polystyrene screen and the sandblasted aluminum film are shown in Fig. 2 (c) and Fig. 2 (d), respectively. The BRDFs of the two scattering samples are compared in Fig. 2 (b). Here the measured angle is the offset to the normal of the diffuse reflectors.

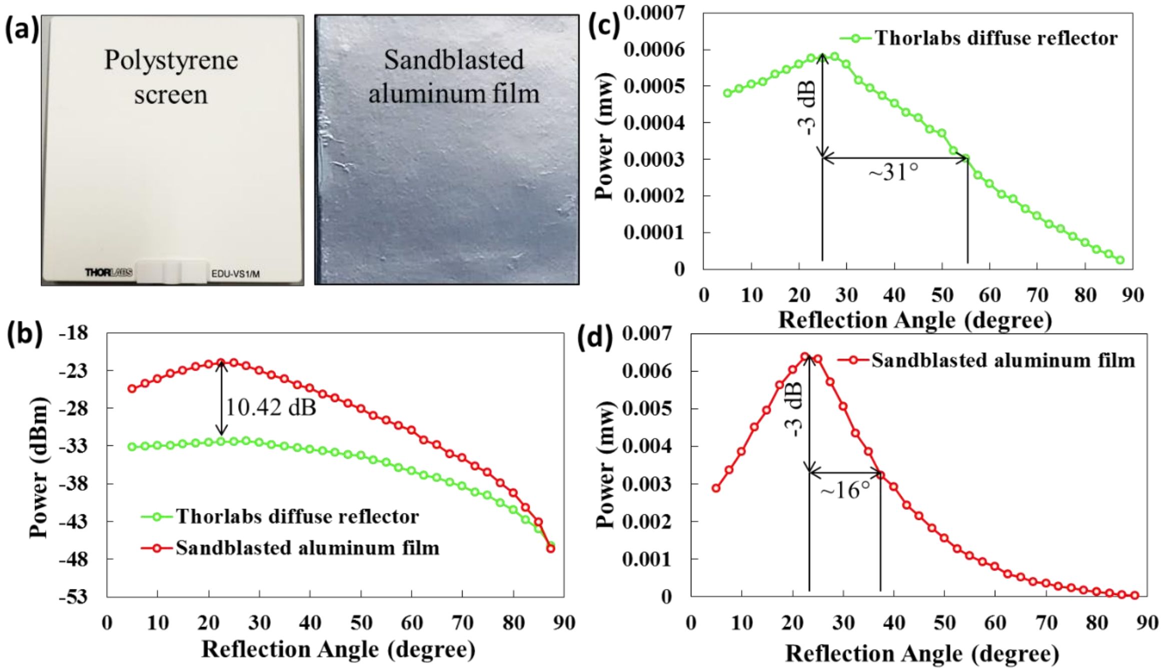

Fig. 2 Reflectors/materials. (a) the photos of the two proposed samples; (b) comparison of the measured BRDFs of both samples in logarithmic scale; (c) and (d) are the measured BDRFs in mw, respectively.

As shown in Fig. 2 (c), the polystyrene screen scatters the diffuse reflected light in a wide range of reflection angles (±31°). However, the received power within this angular range is relatively low (~-33 dBm). In contrast, our sandblasted aluminum sample has a much higher received power (10.42-dB higher at peak point), while sacrificing the angular coverage (±16°). These results demonstrate the tradeoff between the angular coverage and the received power.

S3 Link performance evaluation

In Table-I, the system performance versus time is evaluated. The measurement is performed over three time periods (every 2 seconds) and each contains three Q calculations [2] (calculate every 9.6 μs) at an angular offset of 0°. The results show that the Q factor fluctuates within a very small range of <0.4 dB, which means the system is stable in time. Table-II describes the best performances when receivers are located at 0° and ±10°. When angle offset increases by 10°, transmission performance declines and the Q value is just near the threshold of 3.8×10^{-3} (Q=15.17 dB). Thus the experimentally demonstrated steered angular range is 20°. The constellations at different times and angles are shown in Fig. 3 to further evaluate the system's performance.

Table-I Q factor versus time (0 degree)

Time	Q (SNR)	Time	Q (SNR)	Time	Q (SNR)
0 s	16.02 dB	2 s	16.01 dB	4 s	15.86 dB
0 s+9.6 μs	15.62 dB	2 s+9.6 μs	15.98 dB	4 s+9.6 μs	15.87 dB
0 s+19.2μs	15.66 dB	2 s+19.2 μs	15.79 dB	4 s+19.2 μs	15.83 dB

Table-II Q factor versus angle (0 s)

Angle	Q (SNR)
0º	16.02 dB
10º	15.25 dB
-10º	15.20 dB

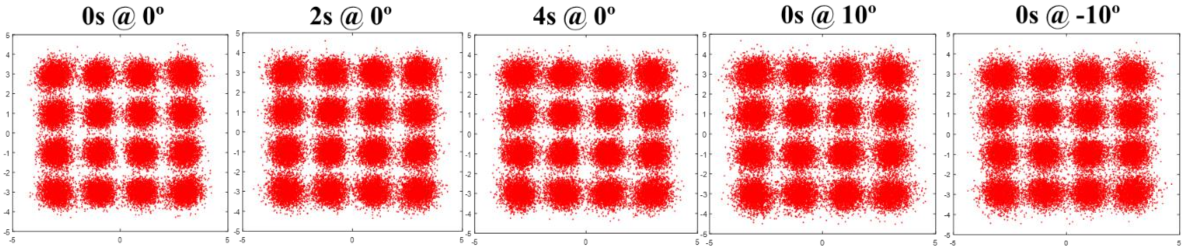

Fig. 3 The constellations at different measurement times and detection angles

Then the Q-to-subcarrier distribution at the time of 0 s is provided in Fig. 4. The subcarriers occupy a frequency range of 9.23 GHz (from -4.615 GHz to 4.615 GHz). Due to the limit of bandwidth of the system and low frequency impacts, the Q factor of some subcarriers is reduced. The system's Q factor is the average value of all subcarriers, which determines the whole transmission capacity. The average Q factor is above the transmission limit of QAM-16 signal.

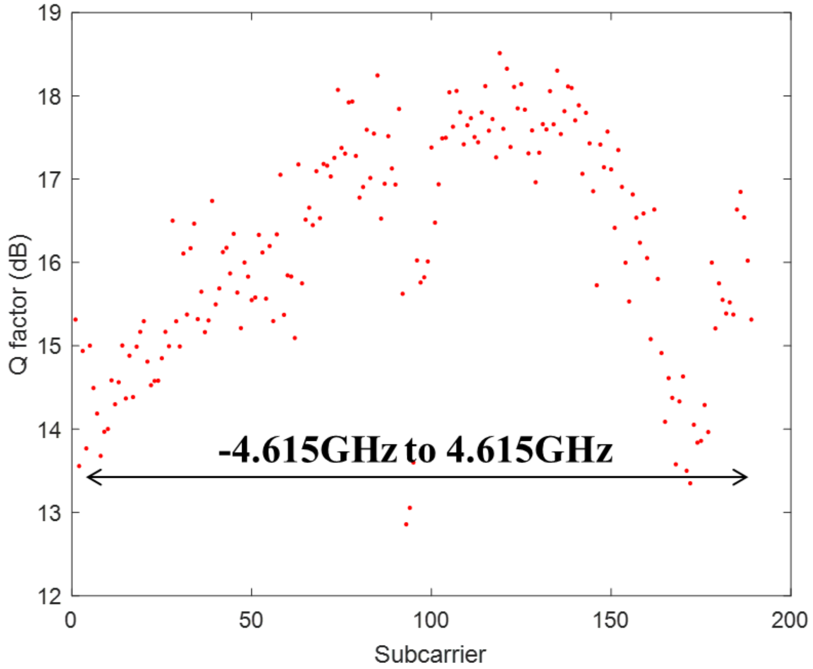

Fig. 4 Q factor versus subcarrier

S4 Additional information of experimental setup

Photo of the free-space link

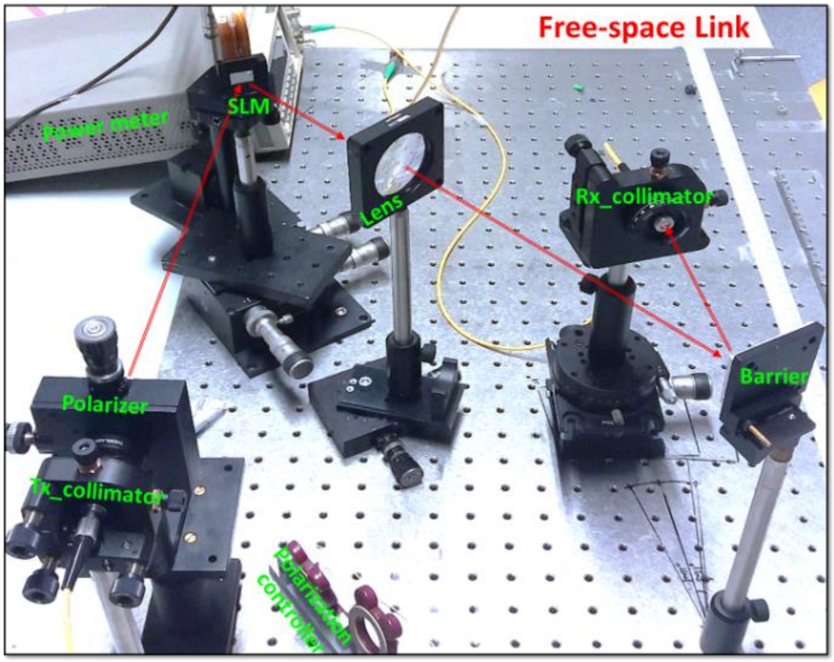

Fig. 5 the photo of the free-space link

Detailed parameters of SLM (Holoeye PLUTO-TELCO)

Type	LCOS (relfective), Active Matrix LCD
Drive scheme	digital (pulse width modulation)
Mode	ECB mode, nematic
Phase level	256 (8-bit) grey levels
Active arera	15.36 mm×8.64 mm
Resolution	
Nominal	1920×1080 pixels
Total	1952×1088 pixels
Pixel pitch	8 μm
Fill factor	87%
Image frame rate	60 Hz
0th order intensity	60%
Illumination (max)	~2 W/cm^2
Operating temperature	+10 ºC -+70 ºC
Front reflection	<0.5%
Reflectivity	80%
Wavelength range	1520 nm-1620 nm
Phase change	0-2π @ 1550 nm

References

[1] W. Shieh, H. Bao, and Y. Tang, "Coherent optical OFDM: theory and design," Optics Express, vol. 16, pp. 841-859, 2008/01/21 2008.
[2] W. Shieh and I. Djordjevic. OFDM for optical communications. Academic Press, 2009.
