# Supplementary material for: Reconfigurable beam system for non-line-of-sight free-space optical communication
Source: Light Sci Appl. 2019 Jul 24;8:69. doi: 10.1038/s41377-019-0177-3 (PMC6804797; doi:10.1038/s41377-019-0177-3)
Supplement: Supplementary file 2 — Authoriaztion from the copyright holder [file 41377_2019_177_MOESM2_ESM.pdf]

IEEE LICENSE
TERMS AND CONDITIONS

Nov 26, 2018

This Agreement between Eindhoven University of Technology -- Xuebing Zhang ("You") and IEEE ("IEEE") consists of your license details and the terms and conditions provided by IEEE and Copyright Clearance Center.

All payments must be made in full to CCC. For payment instructions, please see information listed at the bottom of this form.

License Number	4476411452353
License date	Nov 26, 2018
Licensed Content Publisher	IEEE
Licensed Content Publication	IEEE Proceedings
Licensed Content Title	Non-Line-of-Sight Beam Reconfigurable Optical Wireless System for Energy-Efficient Communication
Licensed Content Author	Z. Cao; X. Zhang; Ivo Vellekoop; A. M. J. Koonen
Licensed Content Date	Oct 29, 0008
Type of Use	Journal/Magazine
Requestor type	non-commercial/non-profit
I am an IEEE member OR the author of this IEEE content.	member
IEEE Member ID	95110093
Format	electronic
Portion	Text extract
In the following language(s)	Original language
Order reference number	
Title of the article	Non-line-of-sight beam reconfigurable optical wireless system for energy-efficient communications
Publication the new article is in	Light: Science & Application
Publisher of the article	Chinese Optical Society and Springer Nature
Author of new article	Zizheng Cao, Xuebing Zhang, Gerwin Osnabrugge, Ivo Vellekoop and A. M. J. Koonen
Expected publication date	Apr 2019
Estimated size of the article (pages)	7
Requestor Location	Eindhoven University of Technology<br>Technische Universiteit Eindhoven<br>P.O. Box 513<br><br>Eindhoven, 5600MB<br>Netherlands<br>Attn: Eindhoven University of Technology
Billing Type	Credit Card
Credit card info	Visa ending in 9449
Credit card expiration	09/2024
Total	**8.82 EUR**
Terms and Conditions	
